# A Probabilistic Model for Reducing Medication Errors

**DOI:** 10.1371/journal.pone.0082401

**Published:** 2013-12-03

**Authors:** Phung Anh Nguyen, Shabbir Syed-Abdul, Usman Iqbal, Min-Huei Hsu, Chen-Ling Huang, Hsien-Chang Li, Daniel Livius Clinciu, Wen-Shan Jian, Yu-Chuan Jack Li

**Affiliations:** 1 Institute of Biomedical Informatics, National Yang-Ming University, Taipei, Taiwan; 2 College of Medicine Science and Technology, Graduate Institute of Biomedical Informatics, Taipei Medical University, Taipei, Taiwan; 3 School of Health Care Administration, Taipei Medical University, Taipei, Taiwan; 4 Bureau of International Cooperation, Department of Health, Taipei, Taiwan; 5 Institute of Translational Medicine, Taipei Medical University, Taipei, Taiwan; 6 Department of Internal Medicine, School of Medicine, College of Medicine, Taipei Medical University, Taipei, Taiwan; 7 Department of Dermatology, Taipei Medical University - Wan Fang Hospital, Taipei, Taiwan; University of Catania, Italy

## Abstract

**Background:**

Medication errors are common, life threatening, costly but preventable. Information technology and automated systems are highly efficient for preventing medication errors and therefore widely employed in hospital settings. The aim of this study was to construct a probabilistic model that can reduce medication errors by identifying uncommon or rare associations between medications and diseases.

**Methods and Finding(s):**

Association rules of mining techniques are utilized for 103.5 million prescriptions from Taiwan’s National Health Insurance database. The dataset included 204.5 million diagnoses with ICD9-CM codes and 347.7 million medications by using ATC codes. Disease-Medication (DM) and Medication-Medication (MM) associations were computed by their co-occurrence and associations’ strength were measured by the interestingness or lift values which were being referred as Q values. The DMQs and MMQs were used to develop the AOP model to predict the appropriateness of a given prescription. Validation of this model was done by comparing the results of evaluation performed by the AOP model and verified by human experts. The results showed 96% accuracy for appropriate and 45% accuracy for inappropriate prescriptions, with a sensitivity and specificity of 75.9% and 89.5%, respectively.

**Conclusions:**

We successfully developed the AOP model as an efficient tool for automatic identification of uncommon or rare associations between disease-medication and medication-medication in prescriptions. The AOP model helps to reduce medication errors by alerting physicians, improving the patients’ safety and the overall quality of care.

## Introduction

Medications are one of the most powerful tools in modern medicine used for the treatment of diseases. Unfortunately, sometimes instead of providing treatment, they can cause considerable harm and even death, especially if prescribing physicians fail to consider relevant patient data and characteristics [1]–[3]. Barach *et al.* reported that nearly 100,000 individuals die per year in the United States due to preventable medical errors, most of which are medication errors [4], [5]. Studies on medication errors during drug administration in surgical units [3], [6], the incidence of medication errors in intensive care units [7], [8], and in pediatric units [2], [9] reveal that in most cases, preventable medication errors cross barriers and reach to patients [2], [7], [9].

Reducing medication errors to increase patient’s safety is crucial to evaluating hospital performance and improving patient outcomes. Wyatt J.C. *et al.* reveals that information technology (IT) boosts clinical leadership for development and procurement in healthcare [10], [11] by improving reliability, quality, medication safety [12], [13], and most importantly reducing prescription errors [3], [5], [13]–[20]. Information Technology also saves hundreds of billions in annual costs by providing automation for ordering, a key process in modern health care. Such processes include CPOE [15] with clinical decision support (CDS), bar-coded medication administration [21], automated dispensing systems (ADS) [8], and dose drug distribution [22]. Bates D.W. *et al*. claims that the key tools for reducing this gap would be information systems which provide decision support to users during decision making, resulting in improved quality of care [23]. Thus, the knowledge-based CDS review can assure that those orders are safe and comply with guidelines [20], [23].

Most knowledge-based systems were implemented for automated methods, statistically developed by experts at a significant cost to maintain assurance and evidence [24]–[26]. In our study, in order to improve the efficiency of detecting medication errors, we used a set of data mining techniques such as frequent item set mining and association rule mining [27]. Being used in a variety of other fields [28]–[30], data mining techniques are also successfully employed in medical informatics applications such as screening tests for preventive care decision support, to locate potentially unknown adverse effects of drugs [31], as well as identifying disease-drug associations in biomedical literature and clinical texts [32]. Wright A. *et al*. used association rule mining to identify clinically accurate associations between medications, laboratory problems [33], and other related studies [34]–[38].

The aim of this study focuses on medication-disease relationships by applying the association rule mining, and using statistical methods to detect medication errors in the computerized physician order entry (CPOE) systems in order to improve patient’s safety.

## Methods

**Figure 1:**
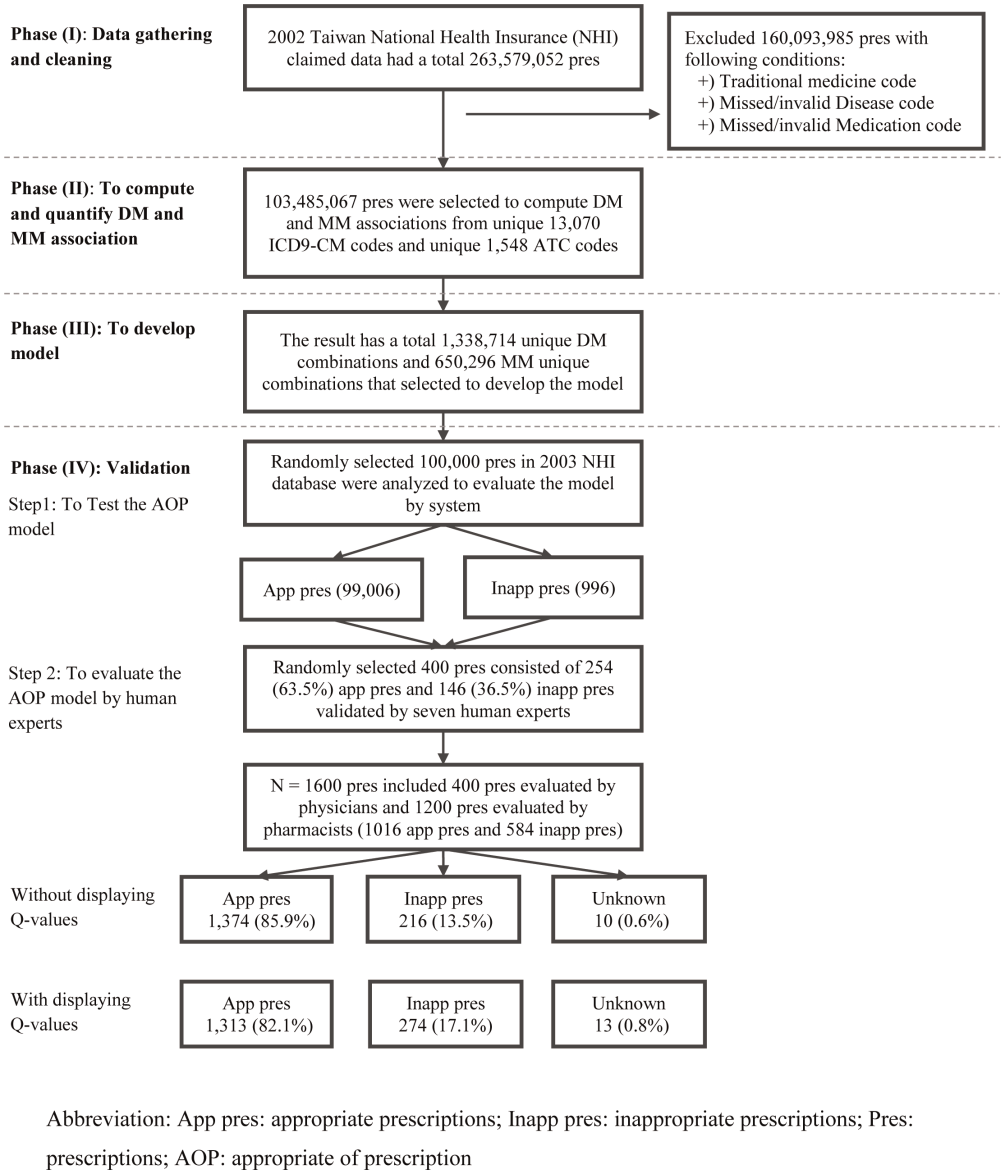
Overall of study design.

In this study, we developed a model to detect uncommon or rare medication for a given disease when ordering prescriptions based on disease-medication associations. The steps involved in methods are described below (see Figure 1):

1. Gathering and cleaning data from the Taiwan National Health Insurance (NHI) database.
2. Quantifying Disease-Medication (DM), Medication-Medication (MM) co-occurrences and computing associations’ strength (Q values) of all the associations present in 103,485,067 prescriptions.
3. Developing a model that can automatically evaluate the **A**ppropriateness **o**f a **P**rescription (AOP).
4. Testing the model for 100,000 randomly selected prescriptions, and then validating the results using seven human experts.

### Phase (I): Data Gathering and Cleaning

The Taiwan’s NHI claims data from Jan 1, 2002 to Dec 31, 2002 with a total of 263.6 million prescriptions from outpatient visits was employed. Each prescription consists of one to three diagnostic codes and one to fifteen medication codes. We excluded 160.1 million prescriptions due to the following reasons: a) missing or invalid disease codes or medication codes, and b) the use of traditional Chinese medicine prescriptions. Thus, the remaining 103.5 million prescriptions with 204.5 million diagnosis ICD9-CM (International Classification of Disease v.9-Clinical Modification) codes and 347.7 million medications with the Taiwan NHI codes were used in the analysis. These medication codes were mapped to the ATC (Anatomical Therapeutic Chemical) classification code system resulting in 13,070 unique ICD9-CM codes and 1,548 unique ATC codes.

### Phase (II): Quantifying Disease-medication (DM), Medication-medication (MM) Co-occurrences and Computing Associations’ Strength (Q Values)

The co-occurrences of disease-medication and medication-medication association were used in our analysis. The data mining techniques used were: 1) Frequent (large) item-set mining and 2) Association rule mining; both are closely related and complementary [33].

1. **Frequent item-set mining** is a technique for locating common items’ co-occurrence in a transaction database [39] and determining possible associations among them.
2. **Association rule mining** is an extension of frequent item-set mining [28], [40], which directs the association between two items in addition to a simple co-occurrence.

Frequent item-set and association rule mining are practical techniques for inferring relationships between disease and medication; thus, many potential rules are often produced through these techniques for filtering, such as a variety of measures for “interestingness” [41], [42]. We termed interestingness or lift value as Q value.

Q value is the ratio between the joint probability of disease-medication and medication-medication with respect to their expected probability under the independent assumption known as lift (interest) and relative risk (RR) in similar studies dealing with associations [33], [41], [43].

**Figure 2:**
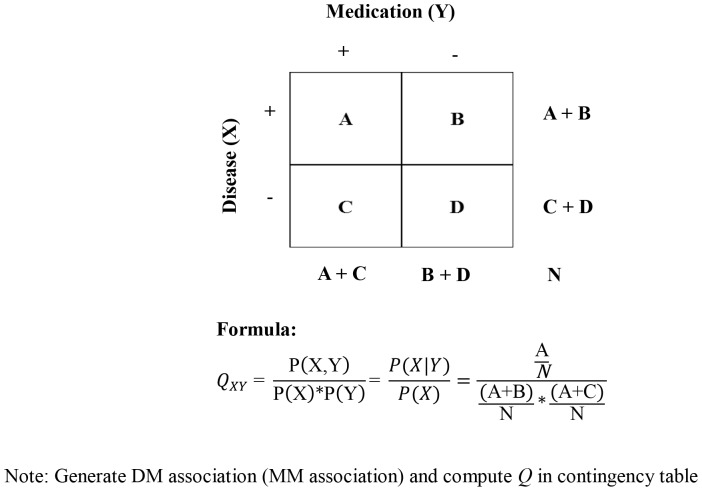
Formulation of Q used in this study.

Furthermore, the disease and medication are considered to be co-occurring if they appeared in the same prescription (see Appendix S1). Based on this definition, each DM pair and MM pair association’s strength was computed using a 2×2 table (Figure 2). Figure 2 shows the equation used to compute the Q value in this study. Q is defined as [0, +∞]; Q = 1 indicates no association between disease and medication, Q <1 indicates that disease and medication are negatively associated (i.e. negative DMQs), and Q >1 indicates that disease and medication are positively associated (i.e. positive DMQs - the prescriptions with disease X containing medication Y occur more often than other medications).

A total of 1.34 million DM and 0.65 million MM pairs with their Q values were computed from 103.5 million prescriptions. The DM and MM associations with less than 5 co-occurrences, by default were considered as “uncommon or rare associations” and were not included in developing the model.

### Phase (III): Developing the Appropriateness of a Prescription (AOP) Model

In this phase, the values of DMQs and MMQs computed in phase (II) are used in developing the AOP model. The Appropriateness of a Prescription (AOP) model is developed based upon following rules:

1. The number of positive DMQs and positive MMQs should be greater than or equal to the number of medications.
2. All diagnoses should have at least one positive DMQ.
3. Each medication should have at least one positive DMQ or positive MMQ.

The AOP model is expressed mathematically as:
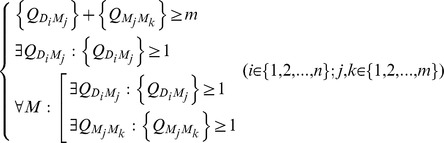
where *n* is the number of diagnoses; *m* is the number of medications; 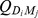 is a DMQ; 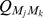 is a MMQ in the same prescription.

The AOP model was built to evaluate associations (DM and MM) present in each prescription and to make a decision whether the overall prescription is appropriate or not. In other words, the model will consider a prescription as appropriate if and only if it has at least one positive DM association present.

### Phase (IV): To Test and Evaluate the AOP Model

Two steps are involved in this phase. First, to test the model based on the verifying dataset and subsequently, evaluating the results by the human experts including four physicians and three clinical pharmacists to measure the accuracy of the AOP model.

#### Step 1: To test AOP model

In the first step, 100,000 prescriptions were randomly selected from the 2003 NHI claims database. Then the AOP model was used to test the selected prescriptions for appropriateness.

#### Step 2: The evaluation of AOP model by human experts

In the second step, 400 prescriptions were randomly selected out of the 100,000 prescriptions and were tested by our AOP model. The 400 prescriptions selected to be evaluated by experts contained 254 (63.5%) appropriate and 146 (36.5%) inappropriate prescriptions. All experts were explained the purpose of the study and were asked to mark whether they agree, disagree or are unsure regarding the overall prescription data provided to them (see Appendix S2). Next, the same prescriptions were re-evaluated with and without the Q values for each DM association present in the prescriptions. We administered the questionnaires (see Appendix S3) to four physicians at their clinic sites (two hundred prescriptions per each physician) and to three clinical pharmacists (eight hundred prescriptions per each pharmacist) at the hospital pharmacies. Overall, we administered 3,200 prescriptions (1,600 prescriptions without Q values and 1,600 with Q values). The average time spent to fill out both questionnaires was about 45 minutes by each physician, and 150 minutes by each pharmacist.

The sensitivity, specificity, positive predictive value (PPV) and negative predictive value (NPV) were computed from the results obtained in order to compare the differences and the consensus between the system and the experts.

## Results

### Results of Step 1

From a total 100,000 prescriptions 99,004 prescriptions (99.004%) were evaluated as appropriate and 996 prescriptions (0.996%) were evaluated as inappropriate by the AOP model.

### Results of Step 2

When the Q values were not disclosed the experts responded to 1590 (99.3%) prescriptions of which 1,374 (85.9%) were appropriate and 216 (13.5%) were inappropriate prescriptions, leaving 10 prescriptions as “unknown”. However, when the Q values were shown in the prescriptions the experts responded only to 1,587 (99.2%) prescriptions of which 1,313 (82.1%) were appropriate, 274 (17.1%) were inappropriate prescriptions, and 13 prescriptions were classified as “unknown” (see Figure 1).

**Figure 3:**
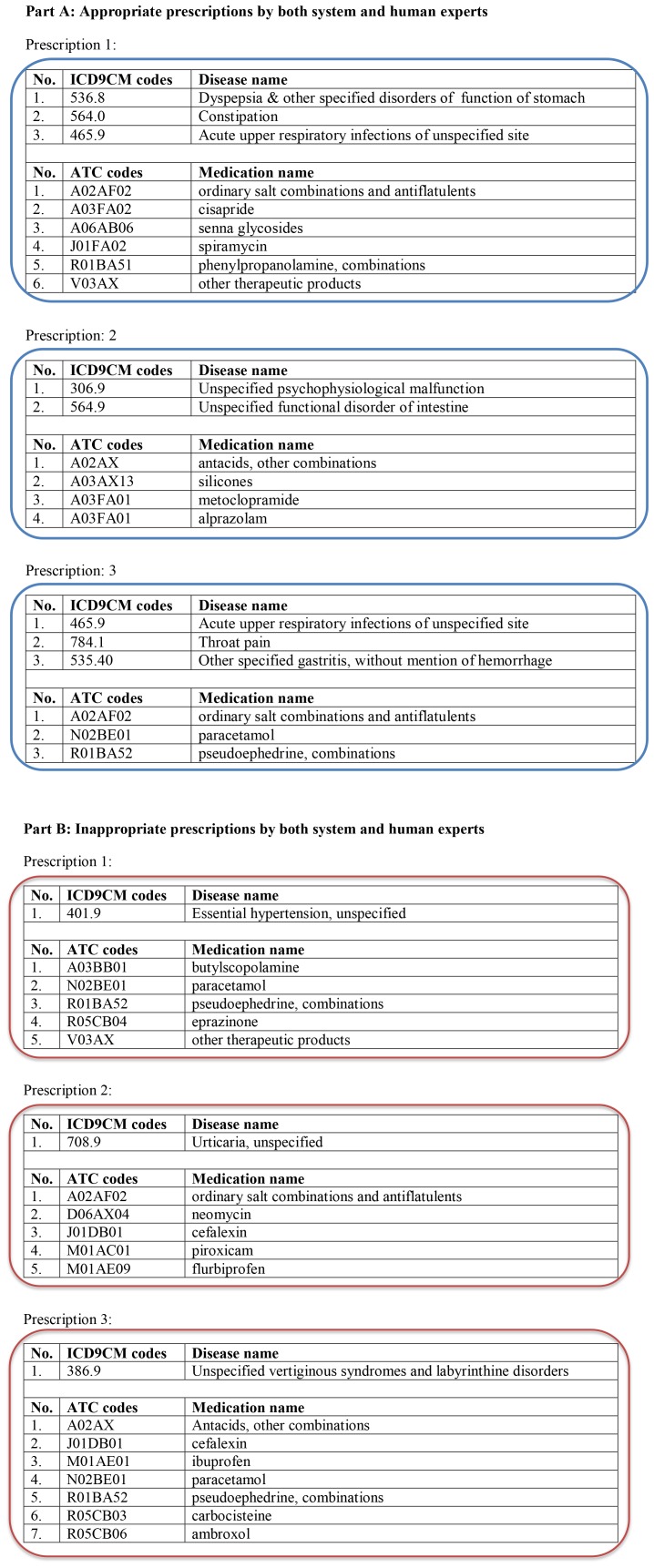
Few examples of the appropriate and inappropriate prescriptions.

The AOP model results were verified by the experts and the sensitivity, specificity, positive (PPV), and negative predictive values (NPV) were computed (see Table 1). While evaluating prescriptions without Q values, we found that the average sensitivity, specificity, PPV, and NPV of the experts were 71.5%, 78.9%, 95.2%, and 33.9%, respectively. However, when Q values were disclosed the average sensitivity, specificity, PPV, and NPV, they were 75.5%, 89.5%, 96.7%, and 45.5%, respectively. A detailed analysis of the results for physicians and clinical pharmacologists versus the AOP model are presented in Table 1. A few examples of the appropriate and inappropriate prescriptions evaluated by both the AOP model and human experts are shown in Figure 3.

**Table 1:** Performance analysis of the results AOP model Vs. human experts (Without and with Q values).

	Without Q values				With Q values			
Human experts	Sens	Spec	PPV	NPV	Sens	Spec	PPV	NPV
Physicians	74.3	82.7	94.8	43.1	76.7	84.9	94.8	50.3
Pharmacists	68.8	75.0	95.6	24.6	74.3	94.2	98.7	40.6
Overall	71.5	78.9	95.2	33.9	75.5	89.5	96.7	45.5

**:** Sens, sensitivity; Spec, specificity; PPV, positive predictive value; NPV, negative predictive value. Note: Confidence intervals (CIs) were small for each parameter and are thus omitted from the reported results. Abbreviation

## Discussion

In this study, we successfully developed, tested, and validated the AOP model, which is able to predict and identify the appropriateness of the prescriptions. The model reveals a high sensitivity and specificity as well as a high positive predictive value, with a negative predictive value around 50%. This AOP model is built on a simple assumption that commonly prescribed medications to a given diseases (DM) are appropriate, and uncommon or rare combinations of disease and medication that might not be appropriate. For example, Sodium Valproate is one of the most effective antiepileptic drugs for suppressing seizure activity; however, it is now well established that Valproate has major teratogenic properties, and when taken during pregnancy can result in more than a 15-fold increased risk of having children with birth defects [44], [45]. Despite Valproate is regarded as one of the commonly used antiepileptic drugs, whenever it is prescribed to the pregnant woman, the AOP will show alert stating it is uncommonly prescribed drug. In addition, we could manually update the software if any new or novel drugs have been added to the hospital formulary. Therefore, the novel therapies that had little or no prior prescribing history would not be further flagged as rare associations.

In this study, we could observe some unexpected strong associations between apparently unrelated disease-medications that we believe were attributable to co-occurrence. For example, let us consider Hypertension as a disease and Insulin as a medication, although Insulin is not directly related with Hypertension; in most prescriptions both hypertension and insulin happen to be present. This is because hypertension has strong association with diabetes. Therefore, Fisher’s exact test with p-value was used to find the significant associations [32], [33]. This additional rule was applied to the AOP model to re-evaluate the 400 prescriptions. An increase in the sensitivity and PPV was observed, however, NPV remained the same. This suggests there is no clear “best” statistical method; instead, selection of the statistical method is dependent on researchers’ preferences and the parameters used for analysis [42], [46]–[48]. In addition, in order to improve the NPV, the cut-off value in the model has to be selected carefully. In this study, the cut-off value for Q is 1. For any association (DM or MM) having Q value less than 1 is regarded as a negative association or uncommon association. The cut-off value can be adjusted to improve NPV.

The AOP model is based only on DMQs and MMQs without having any references or guidelines such as drug-drug interactions, adverse drug events and dose guidance. The validation results of both physicians and pharmacists (PPV) were nearly 96% accurate for appropriate prescriptions and only 45% accurate for inappropriate prescriptions. Van.D.S.H et al. and Taylor.L.K et al. studied the overridden rate to alerts reported from 49% to 96% causing alert fatigue to the Electronic Medical Records (EMR) users [49]–[51]. Compared to their findings, we observed our results show an improved accuracy with a low alert rate given by AOP model, in which only 50% were inappropriate. Thus, if the results were extrapolated to the 0.996% (996) inappropriate prescriptions predicted by this model, the human experts would have considered half of them inaccurate. In this study, we noticed that showing Q valve had no effect on the physicians’ decisions (see Figure S1).

The utilization of association rule mining was reported in several studies such as for relating chief complains and lab results with disorders [33], [52], [53]. To our knowledge, this is the first study using association rule mining techniques to identify the uncommon DM and MM combinations. Based upon it, we developed a model that would able to detect and alert the gaps or medication errors in prescriptions. Thus, our model can significantly help to improve patients’ safety and quality of care in the hospitals.

In summary, the AOP model has a variety of applications. It can be used to alert physicians if medication errors are detected while prescribing medications using the CPOE system. Additionally, the model could be used to reduce the size of medication list in the CPOE for a given diagnosis. An automated medication listing systems and clinical decision support system (CDSS) can also be developed by using the AOP model.

### Limitations

This study has some limitations. We used only 103 million prescriptions to construct our model. It is possible if we analyze 300 or 500 million prescriptions, however, it might affect the association strengths which we obtained by using the original data. Second, only two variables are used, diagnostic code and medication codes in the analysis, however, in the real world the prescription of medications depend on various factors like physician behavior, chief-complains, lab results, and age and gender of patient etc. Third, the seven human experts analyzed only 400 prescriptions in order to evaluate the results we got from our AOP model.

### Conclusion

The AOP model developed in this study is able to detect accurately the inappropriate medications prescribed via COPE system. Thus, the PPV of the validation results from both physicians and pharmacists were accurate for the appropriate prescriptions. Moreover, this model could be applied in clinical practice to aid in improving prescription appropriateness, accuracy, patient safety, and patient care.

## Supporting Information

Figure S1 **Performance statistics for evaluating system by all experts.**
(TIF)

Appendix S1 **An example of prescriptions in raw data.**
(DOCX)

Appendix S2 **Brief Introduction before administering questionnaires.**
(DOCX)

Appendix S3 **Description of questionnaires that used to evaluate the AOP model.**
(DOCX)
