# Supplementary figures and images for: A Probabilistic Model for Reducing Medication Errors
Source: PLoS One. 2013 Dec 3;8(12):e82401. doi: 10.1371/journal.pone.0082401 (PMC3849453; doi:10.1371/journal.pone.0082401)

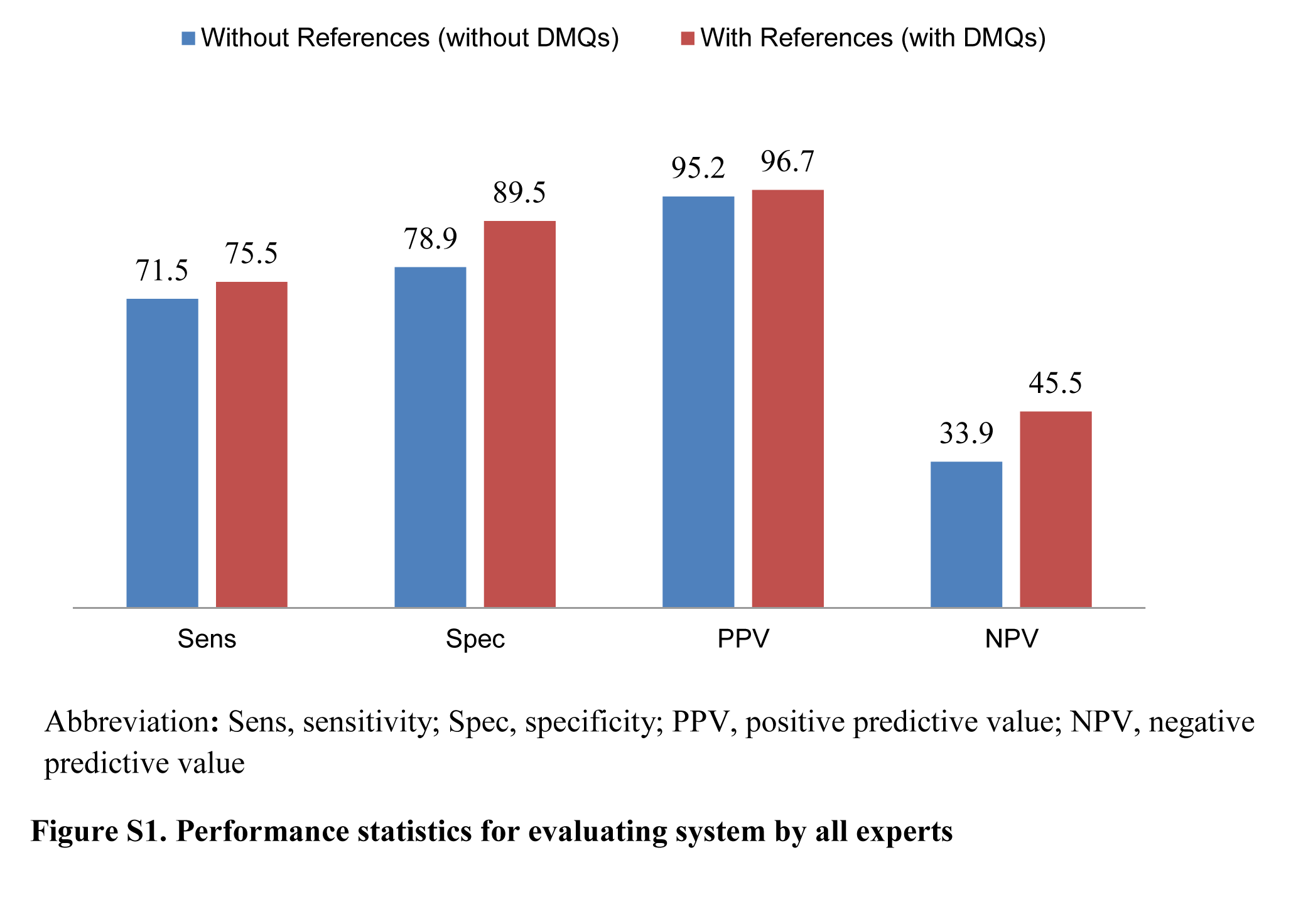

Supplement: Figure S1 — Performance statistics for evaluating system by all experts. (TIF) [file pone.0082401.s001.tif]
