# Supplementary material for: A Probabilistic Model for Reducing Medication Errors
Source: PLoS One. 2013 Dec 3;8(12):e82401. doi: 10.1371/journal.pone.0082401 (PMC3849453; doi:10.1371/journal.pone.0082401)
Supplement: Appendix S1 — An example of prescriptions in raw data. (DOCX) [file pone.0082401.s002.docx]

**Appendix S1. An example of prescriptions in raw data**

| **No.** | **Description of prescription** |
| --- | --- |
| 1 | ‘250.00’; ‘583.9’; ‘455.6’; ‘N02BE01’; ‘M03BB03’; ‘R06AX26’ |
| 2 | ‘044’; ‘006.3’; ‘ ’; ‘J05AE06’; ‘J05AF05’; ‘N05BA12’; ‘A09AA02’; ‘A03AX13’ |
| 3 | ‘401.9’; ‘535.00’; ‘627.9’; ‘C08DB01’; ‘C09AA01’; ‘N05BA06’; ‘C07AA05’; ‘G03DA02’; ‘L02AA91’; ‘G03CA57’; ‘N05CD08’ |
| 4 | ‘455’; ‘ ’; ‘ ’; ‘D11AX’; ‘A03AX96’; ‘A03FA01’; ‘A02AG’; ‘B06AA55’ |
| 5 | ‘386.9’; ‘ ’; ‘ ’; ‘R01BA52’; ‘J01DB01’; ‘A02AX’; ‘R05CB06’; ‘M01AE01’; ‘R05CB03’; ‘N02BE01’ |

- Each row in the table presents each prescription.
- According to Taiwan’s National Health Insurance guidelines, each prescription often consists of:
  - One to three diagnoses with ICD9-CM codes.
  - One to fifteen medication codes with ATC codes.

For example: Prescription 1 contains three ICD9-CM codes and three medications with ATC codes. Prescription 4 contains one ICD9-CM code and seven medications with ATC codes.
