# Supplementary material for: A Probabilistic Model for Reducing Medication Errors
Source: PLoS One. 2013 Dec 3;8(12):e82401. doi: 10.1371/journal.pone.0082401 (PMC3849453; doi:10.1371/journal.pone.0082401)
Supplement: Appendix S2 — Brief Introduction before administering questionnaires. (DOCX) [file pone.0082401.s003.docx]

**Appendix S2. Brief Introduction before administering questionnaires**

**1. Definition of Q:**

For each disease-medication (DM) or medication-medication (MM) association *Q*:

- Q = 1 indicating no association between disease and medication.
- Q < 1 indicating that disease and medication are negatively associated (*negative DMQ*).
- Q > 1 indicating that disease and medication are positively associated (*positive DMQ*).

**2. Rule to administer questionnaires**

Every expert will have three optional choices to fill out the prescription.

- *Option 1*: Please fill the prescriptions “Agree” when you think it is appropriate (or means pharmacists do not need to call physician for intervention).
- *Option 2*: Please fill the prescriptions “Disagree” when you think it is inappropriate (or means pharmacists need to call physician for intervention).
  - For this option, please choose details, which medications you are not agree (or “Disagree” or “Unknown” for each medication).A
- *Option 3*: Please fill the prescription "Agree" when you think hold prescription is appropriate itself, but also could choose details which medications you are not agree (or “Disagree” or “Unknown” for each medication).
  - For this option, the prescription was considered inappropriate when more than 50% medications were filled “Disagree” or “Unknown”.
- *Option 4*: Please fill the prescription "Unknown" when it is not in your knowledge.
