# Supplementary material for: A Probabilistic Model for Reducing Medication Errors
Source: PLoS One. 2013 Dec 3;8(12):e82401. doi: 10.1371/journal.pone.0082401 (PMC3849453; doi:10.1371/journal.pone.0082401)
Supplement: Appendix S3 — Description of questionnaires that used to evaluate the AOP model. (DOCX) [file pone.0082401.s004.docx]

**Appendix S3. Description of questionnaires that used to evaluate the AOP model**

In the question 1, we provided prescriptions with diagnoses codes (ICD9-CM code), diagnoses name (Disease Name) and the medication name (ATC-Drug Name) without references (without showing DMQs) of disease-medication associations. (Example 1)

**Example 1:**

| **ICD9CM_code** | **Disease Name** |
| --- | --- |
| 686.9 | Unspecified local infection of skin and subcutaneous tissue |
| 681.9 | Cellulitis and abscess of unspecified digit |
| 692.9 | Dermatitis unspecified cause |

| **No.** | **ATC Drug Name** | **Agree** | **Disagree** | **Unknown** |
| --- | --- | --- | --- | --- |
| 1 | ordinary salt combinations and antiflatulents |  |  |  |
| 2 | fusidic acid |  |  |  |
| 3 | Flumetasone |  |  |  |
| 4 | Cephalexin |  |  |  |
| 5 | Diclofenac |  |  |  |

In the question 2, we also provided prescriptions with diagnoses codes (ICD9-CM code), diagnoses name (Disease Name) and the medication name (ATC-Drug Name) with references (with showing DMQs) of disease-medication associations. (Example 2)

**Example 2:**

| **ICD9CM_code** | **Disease Name** |
| --- | --- |
| 686.9 | Unspecified local infection of skin and subcutaneous tissue |
| 681.9 | Cellulitis and abscess of unspecified digit |
| 692.9 | Dermatitis unspecified cause |

| **No.** | **ATC Drug Name** | **Agree** | **Disagree** | **Unknown** | **686.9** | **681.9** | **692.9** |
| --- | --- | --- | --- | --- | --- | --- | --- |
| 1 | ordinary salt combinations and antiflatulents |  |  |  | 1.363 | 1.751 | 0.725 |
| 2 | fusidic acid |  |  |  | 32.390 | 26.782 | 8.089 |
| 3 | flumetasone |  |  |  | 4.557 | 5.039 | 21.851 |
| 4 | cephalexin |  |  |  | 4.794 | 8.822 | 0.576 |
| 5 | diclofenac |  |  |  | 1.763 | 1.579 | 0.368 |
